# Association between resting‐state EEG features and cognition in dementia: A community‐based cohort study

**DOI:** 10.1002/alz70855_100115

**Published:** 2025-12-23

**Authors:** Xiaoyan Liang, Xuewen Xiao, Cong Zhang, Sizhe Zhang, Bin Jiao, Lu Shen, Yiliang Liu

**Affiliations:** ^1^ Xiangya Hospital, Central South University, Changsha, Hunan, China

## Abstract

**Background:**

EEG changes have been extensively investigated in neurological disorders. However, it remains unclear whether quantitative differences exist between cognitively normal (CN) and cognitively impaired (CI) elderly populations. Identifying early EEG changes could provide insights into the electrophysiological changes and potential mechanisms underlying pathogenesis of dementia.

**Method:**

3 minutes of resting EEG recordings from all eligible patients were collected using an NVX19 electroencephalograph (Nanjing NeuroMed Technology Group Co., Ltd, China). Patients were instructed to sit relaxed, close their eyes, and avoid falling asleep. Raw EEG data were analyzed using DAFCAR via the NeuroVisor software (Nanjing NeuroMed Technology Group Co., Ltd, China). Cognitive function was assessed with the Mini‐Mental State Examination (MMSE), using education‐adjusted cut‐off scores from the China Population Research Report: illiterate (≤17), primary education (≤20), and junior secondary or higher (≤24). Demographic data, including educational level, medical history, smoking, and alcohol consumption, were also recorded. Additionally, blood samples were collected for *APOE* ε4 genotype sequencing.

**Result:**

A total of 685 elderly individuals over 60 years of age were recruited from the community, including 300 CN and 385 CI participants (Table 1). We analyzed EEG data for peak frequency (PF) and band power across 19 electrodes in various brain regions. Initially, 21 EEG indices exhibited significant differences between the CN and CI groups (*p* < 0.05, Figure 1). After adjusting for age, sex, education, BMI, smoking status, alcohol consumption, related medical history, and APOE ε4 status, 20 indices remained significantly correlated with MMSE scores (Table 2). Specifically, the fronto‐central Alpha correlates negatively with MMSE (F3, C4), whereas temporal Alpha correlates positively (T4, Table 2). Interestingly, Beta1 and Beta2 behave differently at the same site (C3) and across different scalp locations, suggesting it may be important to separate beta into sub‐bands. This pattern suggests a “push–pull” dynamic where Beta1 or Beta2 power can be beneficial in some regions (frontal pole, left temporal, left central) yet detrimental in others (parietal midline, central midline).

**Conclusion:**

Resting state EEG features were significantly different between CN and CI group, and independently associated with cognitive performance.

